# Qualitative Profiling and Quantification of Neonicotinoid Metabolites in Human Urine by Liquid Chromatography Coupled with Mass Spectrometry

**DOI:** 10.1371/journal.pone.0080332

**Published:** 2013-11-12

**Authors:** Kumiko Taira, Kazutoshi Fujioka, Yoshiko Aoyama

**Affiliations:** 1 Department of Anesthesiology, Tokyo Women’s Medical University Medical Center East, Arakawa, Tokyo, Japan; 2 The Hawaii Institute of Molecular Education, Aiea, Hawaii, United States of America; 3 Aoyama Allergy Clinic, Maebashi, Gunma, Japan; Hokkaido University, Japan

## Abstract

Neonicotinoid pesticides have been widely applied for the production of fruits and vegetables, and occasionally detected in conventionally grown produce. Thus oral exposure to neonicotinoid pesticides may exist in the general population; however, neonicotinoid metabolites in human body fluids have not been investigated comprehensively. The purpose of this study is the qualitative profiling and quantitative analysis of neonicotinoid metabolites in the human spot urine by liquid chromatography coupled with mass spectrometry (LC/MS). Human urine samples were collected from three patients suspected of subacute exposure to neonicotinoid pesticides. A qualitative profiling of urinary metabolites was performed using liquid chromatography/time-of-flight mass spectrometry (LC/TOFMS) with a database of nominal molecular weights of 57 known metabolites of three neonicotinoid pesticides (acetamiprid, Imidacloprid, and clothianidin), as well as the parent compounds. Then a quantitative analysis of selected urinary metabolites was performed using liquid chromatography/tandem mass spectrometry (LC/MS/MS) with a standard pesticide and metabolite, which were detected by the qualitative profiling. The result of qualitative profiling showed that seven metabolites, i.e. an acetamiprid metabolite, *N*-desmethyl-acetamiprid; three Imidacloprid metabolites, 5-hydroxy-Imidacloprid, 4,5-dihydroxy-imidacloprid, 4,5-dehydro-Imidacloprid; a common metabolite of acetamiprid and Imidacloprid, *N*-(6-chloronicotinoyl)-glycine; and two clothianidin metabolites, *N*-desmethyl-clothianidin, *N*-(2-(methylsulfanyl)thiazole-5-carboxyl)-glycine, as well as acetamiprid, were detected in the urine of three cases. The result of the quantitative analysis showed *N*-desmethyl-acetamiprid was determined in the urine of one case, which had been collected on the first visit, at a concentration of 3.2 ng/mL. This is the first report on the qualitative and quantitative detection of *N*-desmethyl-acetamiprid in the human urine. The results suggest that the one case with detection of *N*-desmethyl-acetamiprid was exposed to acetamiprid through the consumption of contaminated foods. Urinary *N*-desmethyl-acetamiprid, as well as 5-hydroxy-Imidacloprid and *N*-desmethyl-clothianidin, may be a good biomarker for neonicotinoid exposure in humans and warrants further investigation.

## Introduction

Neonicotinoid pesticides are systemic insecticides that possess nicotinic acetylcholine receptor (nAChR) agonist activity. Especially, imidacloprid causes lethal nicotinic symptoms in mammals [1-6]. Imidacloprid and acetamiprid display excitatory effects on mammalian nAChR at concentrations greater than 1μM, as well as nicotine [7]. Although neonicotinoid pesticides can be environmental neurotoxicants like nicotine, they are used worldwide as agricultural crop protection, environmental pest management and domestic insect control [8]. 

There are several reports on human exposure to neonicotinoid pesticides with analysis of blood or urine samples [1,9-12]; however, qualitative and/or quantitative analysis of neonicotinoid metabolites of human patient’s specimens has rarely been performed, as well as the metabolic pathway of neonicotinoid pesticides in humans. The animal data show numerous toxic and nontoxic metabolites would develop in the brain, plasma, liver and urine after neonicotinoid exposure (Table S1) [13-22]. 

**Figure 1:**
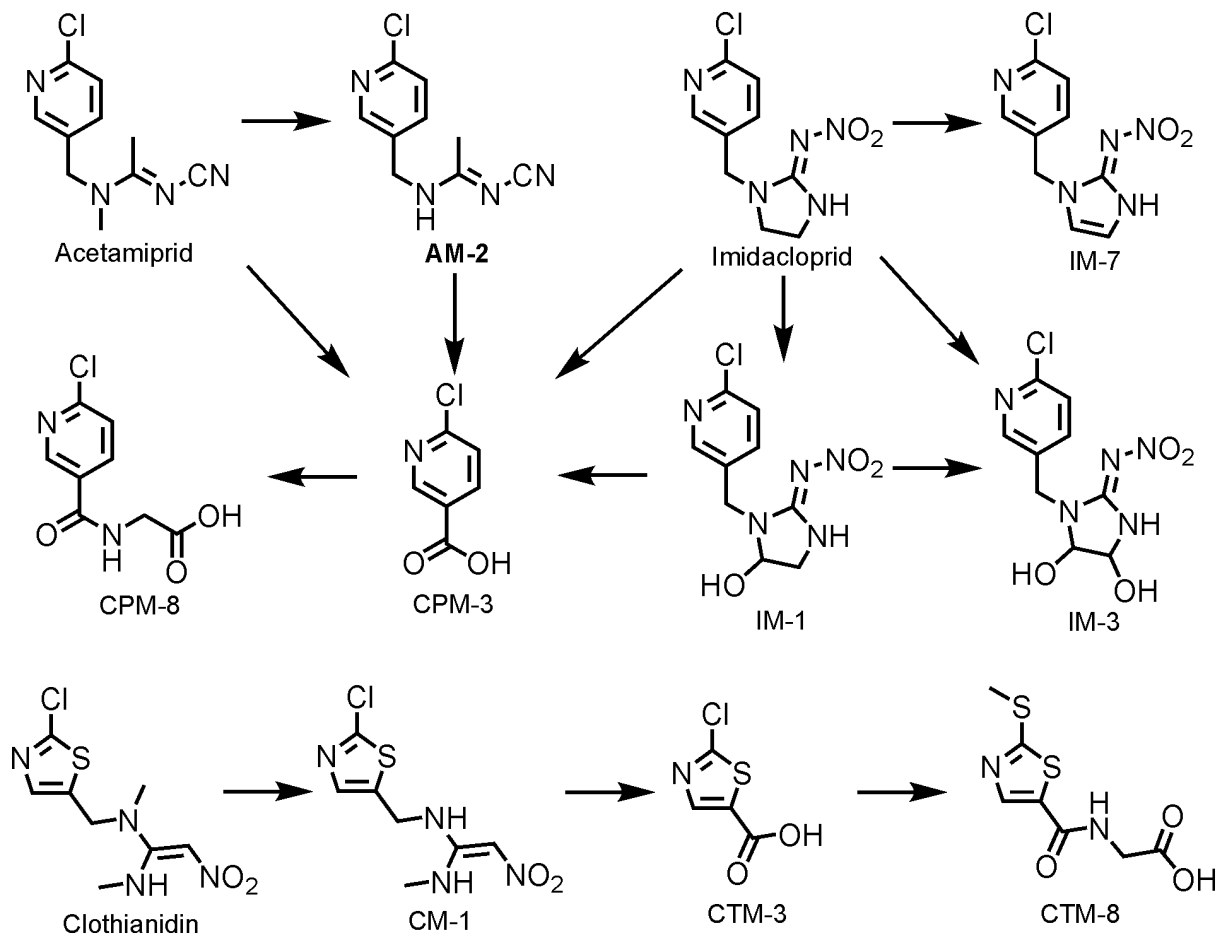
Chemical structures of neonicotinoid insecticides, imidacloprid, acetamiprid and clothianidin, and selected metabolites.

6-Chloronicotinic acid (CPM-3, Figure 1) was found in the urine of patients, who were suspected of subacute exposure of neonicotinoid pesticides, by liquid chromatography/mass spectrometry (LC/MS) and ion chromatography [11]. Although the detection of CPM-3 suggests the exposure of chloropyridinyl neonicotinoid that contains 6-chloropyridinyl moiety, i.e. acetamiprid, imidacloprid, thiacloprid and nitenpyram, it does not point out the identity of exposed pesticides. On the other hand, some neonicotinoid pesticides, i.e. clothianidin, thiamethoxam and dinotefuran, are not metabolized to yield CPM-3, and thus the method is not useful for detecting intoxication or exposure to those compounds [13]. 

*N*-Desmethyl-acetamiprid (AM-2, Figure 1) is reportedly the most dominant urinary metabolite of acetamiprid in rats and a major metabolite in tissues and excreta of ruminants, pigs and poultry after oral administration of acetamiprid in feed for 28 days [23], but this has never been analyzed in human specimens. Therefore conducting a multiple metabolite analysis appears to be promising to monitor the exposure of neonicotinoid pesticides comprehensively and to elucidate their chemical identity. 

Liquid chromatography/time of flight mass spectrometry (LC/TOFMS) has an advantage for detecting unknown chemicals a priori because of its accuracy in the mass separation, and has been used for profiling drug metabolites, as well as screening doping agents [24,25]. We sought to analyze known neonicotinoid metabolites in the spot urine of patients, who were suspected of neonicotinoid pesticide poisoning, using LC/TOFMS qualitatively. As positive controls, urine samples of the mice administered with neonicotinoid pesticides, i.e. acetamiprid, imidacloprid and clothianidin, were used (Figure 1). Then a quantitative analysis of detected substances by LC/TOFMS, i.e. acetamiprid and *N*-desmethyl-acetamiprid (AM-2), in the spot urine of patients, as well as those who didn’t show any typical nicotinic symptoms (negative control group), was conducted using liquid chromatography/tandem mass spectrometry (LC/MS/MS), which has good specificity and high sensitivity [26].

## Materials and Methods

### Materials

Acetamiprid was purchased from Wako Pure Chemical Industries (Osaka, Japan). Imidacloprid, fipronil and formic acid were purchased from Kanto Chemical Co. Inc. (Tokyo, Japan). Clothianidin was purchased from Sigma-Aldrich (St. Louise, MO). An *N*-desmethyl-acetamiprid solution (10 μg/mL) was a gift from Associate Professor Kamata at Kanto Gakuin University. Other chemicals and solvents used were of analytical grade. 

The mouse urine samples were from Professor Ishizuka at Hokkaido University. The mouse urine samples were prepared according to the previously reported method with minor modifications [13,14]. Mice were housed and maintained according to the National Research Council Guide for the Care and Use of Laboratory Animals [27]. All the animal experiments were performed under the supervision and with the approval of the Institutional Animal Care and Use Committee of Hokkaido University (No.10-0004). Male ddY mice (6 months old) were obtained from Japan SLC Inc. (Hamamatsu, Japan) and acclimated for one week in the laboratory. The mice were kept in 40% humidity at 25°C in a temperature-controlled room with a 12 h light/dark cycle. The mice were given laboratory food and clean water ad libitum. Each of the three male ddY mice was intra-peritoneally administered with acetamiprid (10 mg/kg), imidacloprid (10 mg/kg) or clothianidin (20 mg/kg) as solutions of dimethyl sulfoxide. The mice were kept in a metabolic cage for 12 h for urine collection. The urine samples were diluted with the same volume of deionized water and stored at -20 °C until analysis.

 After the approval by the ethics committee of Tokyo Women’s Medical University (No.2810), human urine samples were collected from the patients as spot urine, who visited a clinic in Japan from 2008 to 2012, and written informed consent gave to us. All urine samples were stored at -20 °C until analysis. 

Three urine samples (#1, #2 and #3) were from the patients, in whose urine CPM-3 was detected more than LOQ (2 ng/mL) by LC/MS within several days after the first visit in our previous study [11]. The demographic data and clinical findings of the three patients are shown in Table 1. All patients were non-smokers. They were suspected of subacute intoxication of neonicotinoid pesticides distinguished by four characteristics as follows: i) exhibition of the following symptoms with unknown origin: 4 major symptoms (headache, general fatigue, finger tremor and short-term memory disturbance) and more than four of 6 minor symptoms (fever, cough, palpitation, chest pain, stomachache or muscle pain with muscle spasm) on the first visit; ii) abnormal electrocardiographic findings, e.g. tachycardia, bradycardia, arrhythmia, electrical conduction delay, ST-T change or QT prolongation; iii) high domestic fruit intake (>500 g/day) or high tea beverage intake (>500 mL/day) for more than several days in advance of the first visit; iv) no history of occupational exposure to pesticide. From 2008 to 2012, one hundred and three patients (male/female = 20/83) visited a clinic with these four characteristics. In the urine of the patients who had these four characteristics, 6-chloronicotinic acid (CPM-3) was frequently detected [11]. Two urine samples (#1 and #2) were collected on the first visit. The sample of patient 3 was collected on the 73rd day after the first visit; and the patient had been restricted her intake of fruits and tea beverage after the first visit and recovered from her symptoms. 

**Table 1:** Demographic data and clinical findings of patients, who are suspected of subacute neonicotinoid exposure.

Case	Sex	Age	Electrocardiographic (ECG) findings	Heart rate (bpm)	Intake of fruit and tea beverage before urine sampling	Maximum CPM-3 detection in urine by LC/MS analysis	Total days to need the symptoms and ECG abnormality diminished	The day of urinesampling in this study
#1	F	22	Intermittent WPW syndrome	110	Tea, grapes, Asian pear	59.1 ng/mL (on the 2^nd^ day)	25 days	on the first day of visit
#2	F	27	Sinus tachycardia, QT prolongation	100	Asian pear	6.1 ng/mL (on the 4^th^ day)	8 days	on the first day of visit
#3	F	34	Regular sinus rhythm* (on the 73^rd^ day)	68	None**	12.0 ng/mL (on the 5^th^ day)	43 days	on the 73rd day after the first visit

* Sinus bradycardia (53 bpm), ST-T change at the first visit. ** Tea and Asian pear before the first visit. Data were partially reported in our previous article [11].

Seven human urine samples (#4−10) as the negative controls for LC/MS/MS analysis were collected from the patients who visited a clinic one day without the history of nicotinic symptoms listed above as i). The demographic data of the negative controls are listed in Table 2 (age (mean ± SD): 42.9 ± 9.8 years old; M/F = 3/4).

**Table 2:** Demographic data and the urinary levels of acetemiprid and AM-2 of the negative control group.

**Case**	**Sex**	**Age**	**Acetamiprid**	**AM-2**
			**(ng/mL)**	**(ng/mL)**
#4	F	30	<LOD	0.40 (<LOQ)
#5	F	31	<LOD	<LOD
#6	M	40	<LOD	<LOD
#7	M	40	<LOD	<LOD
#8	F	50	<LOD	<LOD
#9	F	51	<LOD	<LOD
#10	M	58	<LOD	<LOD

The human blank urine was collected from the authors. The authors restricted the intake of conventionally produced fruits and tea beverage before taking urine samples.

### Methods

#### Qualitative profiling of neonicotinoid metabolites by LC/TOFMS

LC/TOFMS analysis, as well as data mining, was performed at a contract research organization (Genaris, Yokohama, Japan) for the mice urine samples and the human urine samples of the three patients (#1, #2 and #3). 

### Sample preparation

The urine samples were thawed, centrifuged at 15,000 rpm for 10 min and filtered with membrane filters (pore size: 0.45 μm). Two types of preparation were performed for the mice urine samples (positive controls) and one for the human samples. Human urine samples were prepared with solid-phase extraction (SPE), because in a preliminary analysis CPM-3 was not observed from the human urine samples, which was prepared with 2-fold dilution without SPE.

a. The positive control was diluted 5-fold without SPE
  The mouse urine was diluted with 5 % acetonitrile/water by 5-fold.
b.The positive control was diluted 5-fold with acidic, neutral or basic SPE
  For neutral SPE, the mixture of 20 μL of the mouse urine and 480 μL of water was loaded in a pre-conditioned SPE cartridge (Plexa, 30 mg, 40 μm, Varian, Palo Alto, CA), washed with 1 mL of water and extracted with 500 μL of acetonitrile. The acetonitrile fraction was concentrated in vacuo for 60 min and reconstructed with 100 μL of 5 % acetonitrile/water containing 0.1 % formic acid. Acidic and basic SPE were performed as the same way except acidic SPE was carried out by adding 1.25 μL of formic acid to the diluted urine sample, and the basic SPE was carried out by adding 10 μL of 25 % ammonium hydroxide to the diluted urine sample.
c. The human urine was concentrated 10-fold with acidic, neutral or basic SPE
  For neutral SPE, 1000 μL of the human urine (#1, #2 and #3) was loaded in a pre-conditioned SPE cartridge (Plexa, 30 mg, 40 μm, Varian, Palo Alto, CA), washed with 1 mL of water and extracted with 500 μL of acetonitrile. Subsequent concentration and reconstruction were performed as described above (b), as well as the acidic/basic SPE except for adding 2.5 μL of formic acid for acidic SPE and 20 μL of 25 % ammonium hydroxide for basic SPE to the human urine.

### Instrumentation

An ACQUITY UPLC (Waters, Milford, MA) equipped with a LCT Premier XE (Waters) and an ACQUITY UPLC HSS T3 column (2.1 x 50 mm, Waters) was used for LC/TOFMS analysis. Mobile phases were water and acetonitrile containing 0.1 % formic acid with a linear gradient. The flow rate was 0.4 mL/min. Ionization modes were both positive and negative. The range of detection was from *m*/*z* = 50 to 1000. The volume of injection was 5 μL for positive controls and 10 μL for human urine samples (#1, #2 and #3). The LC/TOFMS analysis was carried out twice for each sample; and mean intensities were obtained for both positive and negative modes of ionization.

### Data analysis

Detection of neonicotinoid and metabolites in the positive control: Data mining was performed using metaProfiling and metaComparing software (Genaris) with four sets of mass spectra, including two sets of data for each ionization mode, for each urine sample and the database of neonicotinoid pesticides, known metabolites and biological chemicals. The cut off threshold for mass was set at ± 20 ppm. The limit of quantification (LOQ) was defined as a signal/noise ratio of 10 and estimated to be 100 arbitrary units. A comparison analysis was performed with the selected peaks in the three positive controls for identification of neonicotinoid and metabolites. The previously known 57 metabolites were studied, which might develop in the positive control mouse urine by acetamiprid, imidacloprid, or clothianidin [13-15]. The nominal molecular weights of neonicotinoids and metabolites used in this study are listed in Table 3. Those 57 metabolites used in this study can be categorized into six classes, that is, 12 are unique for acetamiprid (AM-1–12), 11 are unique for imidacloprid (IM-1–11), 13 are unique for clothianidin (CM-1–13), 10 are common for chloropyridinyl neonicotinoid (CPM-1–10), one is common for imidacloprid and clothianidin (ICM-1), and 10 are common for clothianidin and thiamethoxam (CTM-1–10) (Table 3, Table S1). Although CTM-1−10 can be developed from thiamethoxam directly, CM-1−13 is always developed from clothianidin, which can be developed from thiamethoxam. False positive detections were ruled out by comparing the detection pattern with positive controls. In addition, multiple peaks derived from an identical peak with tailing, e.g. 42 peaks for clothianidin, were inspected with chromatograms, and the strongest peak with the earliest retention time was assigned as a parent peak. Identification of detected peaks was carried out by comparison with the literature values of retention time [13,14]. Optionally, isotopic pattern of chlorine-35/37 was also used for the confirmation whenever it is available. 

**Table 3:** The names, IUPAC names, molecular formula and molecular masses of three neonicotinoid pesticides and metabolites used in this study.

**Name**	**IUPAC Name**	**Formula**	**Mass (amu)**
Acetamiprid	(E)-N1-[(6-chloro-3-pyridyl)methyl]-N2-cyano-N1-methylacetamidine	C_10_H_11_ClN_4_	222.0672
AM-1	N-[(6-chloropyridin-3-yl)methyl]-N-methylacetamide	C_9_H_11_ClN_2_O	198.0560
AM-2	(E)-N1-[(6-chloro-3-pyridyl)methyl]-N2-cyano-acetamidine	C_9_H_9_ClN_4_	208.0516
AM-3	N-[(6-chloropyridin-3-yl)methyl]acetamide	C_8_H_9_ClN_2_O	184.0403
AM-4	(E)-N1-[(6-chloro-3-pyridyl)methyl]-N2-carbamoylacetamidine	C_9_H_11_ClN_4_O	226.0621
AM-5	(E)-N1-[(6-chloro-3-pyridyl)methyl]-N2-(acetylcarbamoyl)-acetamidine	C_11_H_13_ClN_4_O_2_	268.0727
AM-6	1-(6-chloropyridin-3-yl)-N-methylmethanamine	C_7_H_9_ClN_2_	156.0454
AM-7	(6-chloropyridin-3-yl)methanamine	C_6_H_7_ClN_2_	142.0298
AM-8	N-[(6-chloropyridin-3-yl)methyl]formamide	C_7_H_7_ClN_2_O	170.0247
AM-9	(E)-N2-cyano-N1-methylacetamidine	C_4_H_7_N_3_	97.0640
AM-10	(E)-N1-[(2S,3S,4S,5R,6R)-2-carboxy-3,4,5-trihydroxyoxan-6-yl]-N2-cyano-N1-acetamidine	C_10_H_15_N_3_O_6_	273.0961
AM-11	N2-cyanoacetamidine	C_3_H_5_N_3_	83.0483
AM-12	N1-[(6-chloropyridin-3-yl)methyl]-N1-methylacetamidine	C_9_H_12_ClN_3_	197.0720
Imidacloprid	1-(6-chloro-3-pyridylmethyl)-N-nitroimidazolidin-2-ylideneamine	C_9_H_10_ClN_5_O_2_	255.0523
IM-1	1-(6-chloro-3-pyridylmethyl)-N-nitro-5-hydroxyimidazolidin-2-ylideneamine	C_9_H_10_ClN_5_O_3_	271.0472
IM-2	1-(6-chloro-3-pyridylmethyl)-2-nitroguanidine	C_7_H_8_ClN_5_O_2_	229.0367
IM-3	1-(6-chloro-3-pyridylmethyl)-N-nitro-4,5-dihydroxyimidazolidin-2-ylideneamine	C_9_H_10_ClN_5_O_4_	287.0421
IM-4	1-(6- chloro-3-pyridylmethyl)imidazolidin-2-ylideneamine	C_9_H_11_ClN_4_	210.0672
IM-5	1-(6-chloro-3-pyridylmethyl)-Naminoimidazolidin-2-ylideneamine	C_9_H_12_ClN_5_	225.0781
IM-6	1-(6-chloro-3-pyridylmethyl)-N-nitrosoimidazolidin-2-ylideneamine	C_9_H_10_ClN_5_O	239.0574
IM-7	1-(6- chloro-3-pyridylmethyl)-N-nitroimidazolin-2-ylideneamine	C_9_H_8_ClN_5_O_2_	253.0367
IM-8	N5-[1-[(6-chloropyridin-3-yl)methyl]-2-methyl-1,2,6,7-tetrahydro-imidazo[1,2-b][1,2,4]triazin-1-one	C_12_H_12_ClN_5_O	277.0730
IM-9	1-(6-chloropyridin-3-ylmethyl)imidazolin-2-one	C_9_H_10_ClN_3_O	211.0512
IM-10	N-nitroimidazolidin-2-ylideneamine	C_3_H_6_N_4_O_2_	130.0491
IM-11	N-nitroimidazolin-2-ylideneamine	C_3_H_4_N_4_O_2_	128.0334
Clothianidin	(E)-1-(2-chloro-1,3-thiazol-5-ylmethyl)-3-methyl-2-nitroguanidine	C_6_H_8_ClN_5_O_2_S	249.0087
CM-1	(E)-1-(2-chloro-1,3-thiazol-5-ylmethyl)-2-nitroguanidine	C_5_H_6_ClN_5_O_2_S	234.9931
CM-2	1-(2-chloro-1,3-thiazol-5-ylmethyl)-guanidine	C_5_H_7_ClN_4_S	190.0080
CM-3	2-amino-1-(2-chloro-1,3-thiazol-5-ylmethyl)-guanidine	C_5_H_8_ClN_5_S	205.0189
CM-4	(E)-1-(2-chloro-1,3-thiazol-5-ylmethyl)-2-nitrosoguanidine	C_5_H_6_ClN_5_OS	218.9982
CM-5	3-(2-chloro-1,3-thiazol-5-ylmethylamino)-6-methyl-4,5-dihydro-1,2,4-triazine-5-one	C_8_H_8_ClN_5_OS	257.0138
CM-6	1-(2-chloro-1,3-thiazol-5-ylmethyl)-urea	C_5_H_6_ClN_3_OS	190.9920
CM-7	1-(2-chloro-1,3-thiazol-5-ylmethyl)-3-methylguanidine	C_6_H_9_ClN_4_S	204.0236
CM-8	2-amino-1-(2-chloro-1,3-thiazol-5-ylmethyl)-3-methylguanidine	C_6_H_10_ClN_5_S	219.0345
CM-9	(E)-1-(2-chloro-1,3-thiazol-5-ylmethyl)-3-methyl-2-nitrosoguanidine	C_6_H_8_ClN_5_OS	233.0138
CM-10	3-(2-chloro-1,3-thiazol-5-ylmethylamino)-4,6-dimethyl-4,5-dihydro-1,2,4-triazine-5-one	C_9_H_10_ClN_5_OS	271.0295
CM-11	1-(2-chloro-1,3-thiazol-5-ylmethyl)-3-methylurea	C_6_H_8_ClN_3_OS	205.0077
CM-12	2-methyl-1-nitroguanidine	C_2_H_6_N_4_O_2_	118.0491
CM-13	2-methylguanidine	C_2_H_7_N_3_	73.0640
CPM-1	6-chloropyridine-3-carbaldehyde	C_6_H_4_ClNO	140.9981
CPM-2	(6-chloropyridin-3-yl)methanol	C_6_H_6_ClNO	143.0138
CPM-3	6-chloropyridine-3-carboxylic acid	C_6_H_4_ClNO_2_	156.9931
CPM-4	6-(6-chloropyridine-3-carbonyl)-(2S,3S,4S,5R,6R)-3,4,5,6-tetrahydroxyoxane-2-carboxylic acid	C_12_H_12_ClNO_8_	333.0251
CPM-5	S-(3-carboxypyridin-6-yl)-(2R)-2-acetamido-3-sulfanyl-propanoic acid	C_11_H_12_N_2_O_5_S	284.0467
CPM-6	6-methylsulfanylpyridine-3-carboxylic acid	C_7_H_7_NO_2_S	169.0197
CPM-7	6-oxo-1H-pyridine-3-carboxylic acid	C_6_H_5_NO_3_	139.0269
CPM-8	2-[(6-chloropyridine-3-carbonyl)amino]acetic acid	C_8_H_7_ClN_2_O_3_	214.0145
CPM-9	2-[(6-(methylsulfanyl)pyridine-3-carbonyl)amino]acetic acid	C_9_H_10_N_2_O_3_S	226.0412
CPM-10	6-sulfonyloxypyridine-3-carboxylic acid	C_6_H_5_NO_6_S	218.9838
ICM-1	2-nitroguanidine (1-nitroguanidine)	CH_4_N_4_O_2_	104.0334
CTM-1	2-chloro-1,3-thiazole-5-carbaldehyde	C_4_H_2_ClNOS	146.9546
CTM-2	(2-chloro-1,3-thiazol-5-yl)methanol	C_4_H_4_ClNOS	148.9702
CTM-3	2-chloro-1,3-thiazole-5-carboxylic acid	C_4_H_2_ClNO_2_S	162.9495
CTM-4	6-(2-chloro-1,3-thiazole-5-carbonyl)-(2S,3S,4S,5R,6R)-3,4,5,6-tetrahydroxyoxane-2-carboxylic acid	C_10_H_10_ClNO_8_S	338.9816
CTM-5	S-(5-carboxy-1,3-thiazol-2-yl)-(2R)-2-acetamido-3-sulfanyl-propanoic acid	C_9_H_10_N_2_O_5_S_2_	290.0031
CTM-6	2-methylsulfanyl-1,3-thiazole-5-carboxylic acid	C_5_H_5_NO_2_S_2_	174.9762
CTM-7	2-[(2-chloro-1,3-thiazole-5-carbonyl)amino]acetic acid	C_6_H_5_ClN_2_O_3_S	219.9709
CTM-8	2-[(2-methylsulfanyl-1,3-thiazole-5-carbonyl)amino]acetic acid	C_7_H_8_N_2_O_3_S_2_	231.9976
CTM-9	(2-chloro-1,3-thiazol-5-yl)methanamine	C_4_H_5_ClN_2_S	147.9862
CTM-10	N-[(2-chloro-1,3-thiazol-5-yl)methyl]acetamide	C_6_H_7_ClN_2_OS	189.9968

AM is a metabolite unique for acetamiprid; IM for imidacloprid; and CM for clothianidin. CPM is a common metabolite for chloropyridinyl neonicotinoid; ICM for imidacloprid and clothianidin; and CTM for clothianidin and thiamethoxam.

Detection of neonicotinoid and metabolites in human urine: Regarding peaks in the human urine, data mining and analysis was conducted with four sets of mass spectra for each human urine sample data combined with positive controls and the database of neonicotinoid pesticides, known metabolites and biological chemicals. A decision criterion was that a substance is detected when one or more signal(s) exceed LOQ of 100 arbitrary units. The cut off threshold for mass was set at ± 20 ppm. The cut off threshold for retention time (*t*_R_) was set at ± 3 seconds (0.05 ppm) for the detected metabolites in positive controls. 

#### Quantification of neonicotinoid metabolites by LC/MS/MS

LC/MS/MS analysis was performed at the Graduate School of Veterinary Medicine, at Hokkaido University (Sapporo, Japan) for the urine samples from three patients (#1,#2 and #3) and negative controls (#4−10). Quantification of neonicotinoid metabolites was performed only for AM-2, because standard compounds were not available for imidacloprid or clothianidin, i.e. IM-1, IM-3, IM-7 or CM-1. Quantification of acetamiprid was also performed at the same time.

### Preparation of urine samples

Standard urine solutions were prepared with blank urine and the working standard solutions of acetamiprid at 10 ng/mL and AM-2 at 100 ng/mL. The lowest concentrations of standard solution for acetamiprid and AM-2 were set based on the approximately 10-fold difference in their instrumental detection limits. The concentrations for acetamiprid and AM-2 were set at 0.05, 0.1, 0.15, 0.2 and 0.25 ng/mL and at 0.5, 1.0, 1.5, 2.0 and 2.5 ng/mL, respectively. 

Fifty μL of 20 ng/mL fipronil solution in 3% acetonitrile/water was added as internal standard (I.S.) to each 1 mL of standard urine solutions and the human urine samples (#1−10) before the SPE treatment.

### Sample preparation by SPE

Urine samples were subjected to SPE and concentrated 10-fold. One mL of the human urine containing 1 ng/mL of fipronil as I.S. was loaded in a pre-conditioned SPE cartridge (Oasis WAX Plus, 225 mg, 60 μm, Waters), washed with 2 ml of 0.1 M NaOH, 50 mM sodium phosphate buffer (pH = 7.4) and water, and extracted with 2 mL of acetonitrile. The acetonitrile fraction was concentrated in vacuo for 60 min and reconstructed with 100 μL of 3 % acetonitrile/water.

### Instrumentation

A Prominence UFLC (Shimadzu, Kyoto, Japan) equipped with an LCMS-8030 (Shimadzu) was used for LC/MS/MS analysis. With respect to a LC column, we used a C18 column (100 Å, 2.6 µm, 2.1 x 50 mm, Phenomenex, Torrance, CA) first. The retention times for AM-2 and acetamiprid by the C18 column were 3.4 and 3.6 min, respectively; however, it did not show a baseline separation of AM-2 from the background noise. In order to obtain a good resolution, we tried a Luna PFP (2) column (100 Å, 2.1 x 50 mm, Phenomenex) next. The retention times for AM-2 and acetamiprid by the PFP column were 3.8 and 4.0 min, respectively; and it showed a better peak separation of AM-2 from the background noise than the C18 column. Consequently, the PFP column was chosen for the further analysis. Mobile phases were water (A) and acetonitrile (B) containing 0.1 % formic acid with a linear gradient from 3% B to 100 % B in 8 min. Flow rate was 0.4 mL/min. The ionization mode was positive with multiple reaction monitoring (MRM) mode. The ion species for quantification of acetamiprid, AM-2 and fipronil were *m*/*z* = 222.80/126.10, *m*/*z* = 209.00/126.10 and *m*/*z* = 435.00/330.00, respectively. The MRM transitions for confirmation of AM-2, acetamiprid and fipronil were *m*/*z* = 209.00/168.20, *m*/*z* = 222.80/141.10 and *m*/*z* = 435.00/250.00, respectively. The collision energies (CE) and other MS parameters were optimized and are shown in Table 4. The nebulizing gas was 3 L/min, the drying gas was 20 L/min, the desolvation line (DL) temperature was 300 °C, and the heat block temperature was 450 °C. The volume of injection was 5 μL. The column temperature was set at 45 °C. Recovery efficiency test was performed with three injections of the extracts of standard urine solution containing 0.1 ng/mL of acetamiprid and 1.0 ng/mL of AM-2.

**Table 4:** The MRM parameters for LC/MS/MS.

**Name**	**Ionization mode**	**MRM transitions (*m/z*)**	**Dwell time (ms)**	**Q1 pre bias (V)**	**CE**	**Q3 pre bias (V)**
AM-2	Positive	209.00/126.10	100	-23.0	-18.0	-19.0
Acetamiprid	Positive	222.80/126.10	100	-24.0	-21.0	-20.0
Fipronil (I.S.)	Negative	435.00/330.00	100	21.0	16.0	22.0

### Statistical analysis

The t-test was used for the statistical data analysis and the level of significant was set at p-value of 0.05.

## Results

### Qualitative profiling of neonicotinoid metabolites in the positive controls

**Figure 2:**
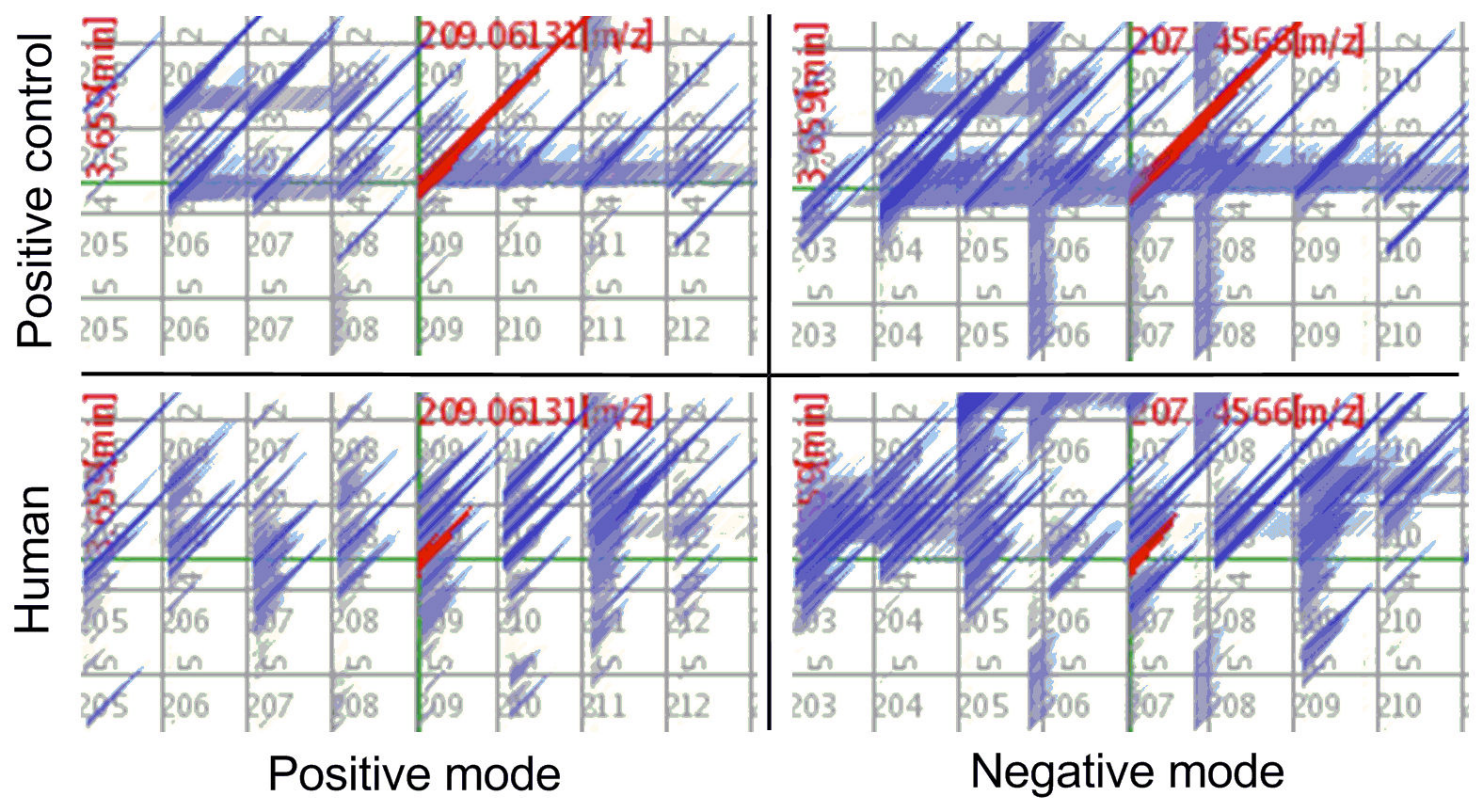
Representative mass chromatograms of LC/TOFMS for *N*-desmethyl-acetamiprid (AM-2) (red): The X-axis is molecular weight and the Y-axis is retention time. The upper row is for the positive control and the lower row is for the human urine (#1). The left column is positive mode and the right column is negative mode.

Thirty-six out of 60 nominated substances were detected in the positive controls by the automatic screening. False positive peaks were discriminated by comparing the detection pattern with three positive controls and peaks derived from tailing, resulting in an identification of 27 substances in the positive controls. The intensity and retention times of detected substances are listed in Table 5. A comparison of retention times of detected peaks with the literature values of HPLC/DAD analysis was carried out [13,14], because they used a reversed-phase C18 column with a gradient of acetonitrile/water containing 0.1 % trifluoroacetic acid, which is comparable to our conditions. The retention time obtained in our study is positively correlated with those studies (n = 19, r^2^ = 0.8937, p < 0.001). Among the 27 substances, four are unique for acetamiprid (acetamiprid and AM-2, 6, 8), seven are unique for imidacloprid (imidacloprid and IM-1, 2, 3, 6, 7, 10), eight are unique for clothianidin (clothianidin and CM-1, 2, 4, 6, 7, 9, 11), four are common for clothianidin and thiamethoxam (CTM-3, 5, 7, 8), and four are common for chloropyridinyl neonicotinoid (CPM-3, 5, 8, 9). Among the 27 substances, 21 showed higher intensities in the positive mode than the negative mode. Representative chromatograms for AM-2 in the positive controls are shown in Figure 2.

**Table 5:** The names, molecular weights, retention times and mean intensities of neonicotinoid pesticide metabolites detected in the urine of positive controls by LC/TOFMS analysis.

**Name**	**Mw**	**t_R_ [min]**	t_R_ [min] in literature**	**Positive mode**				**Negative mode**				Intensity Ratio (%)***		
				**w/o SPE**	**SPE Acid**	**SPE Neutral**	**SPE Base**	**w/o SPE**	**SPE Acid**	**SPE Neutral**	**SPE Base**	**SPE Acid**	**SPE Neutral**	**SPE Base**
Acetamiprid	222.0672	3.877	19.8	230990	136437	143314	130993	178	99	168	122	59.1	62.0	56.7
AM-2*	208.0516	3.661	17.2	176808	103563	108609	102552	166409	148522	184972	178696	58.6	61.4	58.0
AM-6	156.0454	1.185	6.6	195	0	210	1351	0	0	0	0	0.0	107.7	692.8
AM-8	170.0247	3.049	11.2	1195	755	0	0	2436	2245	0	0	63.2	0.0	0.0
CPM-3*	156.9931	3.502	18.0	7305	4483	0	0	15181	13093	5	0	61.4	0.0	0.0
CPM-5	284.0467	3.176	-	28196	13626	254	403	9165	8379	5	0	48.3	0.9	1.4
CPM-8*	214.0145	3.049	12.5	50573	36108	558	572	68100	69922	1851	3009	71.4	1.1	1.1
CPM-9	226.0412	3.315	16.4	17512	11118	3109	891	3680	5049	1783	580	63.5	17.8	5.1
Imidacloprid	255.0523	3.731	18.6	126313	88353	103182	101535	4146	3301	6763	11346	69.9	81.7	80.4
IM-1*	271.0472	3.436	16.1	323966	217876	257912	248064	114805	105583	236919	270837	67.3	79.6	76.6
IM-2	229.0367	3.311	14.9	885	602	1036	3488	3883	4898	10307	16542	68.0	117.1	394.1
IM-3*	287.0421	3.326	15.6	15485	9709	12321	9423	15399	12939	7447	5235	62.7	79.6	60.9
IM-6	239.0574	3.284	13.8	2659	2013	1995	1864	272	1694	855	245	75.7	75.0	70.1
IM-7*	253.0367	3.346	15.2	46911	32384	40533	31812	106791	136173	209794	180295	69.0	86.4	67.8
IM-10	130.0491	0.787	3.5	2138	44	83	109	0	0	0	0	2.1	3.9	5.1
Clothianidin	249.0087	3.617	17.5	546703	406273	430457	455068	238627	487855	526516	560199	74.3	78.7	83.2
CM-1*	234.9931	3.414	16.1	173072	124357	171008	175642	96615	186400	254871	279576	71.9	98.8	101.5
CM-2	190.0080	1.699	-	7915	10217	11728	818	12416	1336	1570	17	129.1	148.2	10.3
CM-4	218.9982	2.747	-	4423	3542	3494	3514	532	827	2812	2827	80.1	79.0	79.4
CM-6	190.9920	3.415	-	2719	1921	2581	2545	0	0	0	0	70.7	94.9	93.6
CM-7	204.0236	2.519	9.6	5121	8169	10597	1031	145	0	0	0	159.5	206.9	20.1
CM-9	233.0138	3.008	12.1	7476	5181	5618	6166	3970	2618	2084	2470	69.3	75.1	82.5
CM-11	205.0077	3.104	12.9	1885	1874	2686	3291	0	0	0	0	99.4	142.5	174.6
CTM-3*	162.9495	3.222	-	314	145	0	0	1059	1099	0	0	46.2	0.0	0.0
CTM-5	290.0031	2.959	-	13305	6936	0	0	1789	746	0	0	52.1	0.0	0.0
CTM-7	219.9709	3.201	-	276	240	0	0	2515	2595	18	0	87.0	0.0	0.0
CTM-8*	231.9976	3.301	-	19958	13989	4081	1332	24310	20448	2156	6326	70.0	20.4	6.7

* Common names of AM-2, IMI-1, IMI-3, IMI-7, CM-1, CPM-3, CPM-8, CTM-3 and CTM-8 are *N*-desmethyl-acetamiprid, 5-hydroxy-imidacloprid, 4,5-dihydroxy-imidacloprid, 4,5-dehydro-imidacloprid, *N*-desmethyl-clothianidin, 6-chloronicotinic acid, *N*-(6-chloronicotinoyl)-glycine, 2-chlorothiazole-5-carboxylic acid and *N*-(2-(methylsulfanyl)thiazole-5-carboxyl)-glycine, respectively.

** the data of HPLC/DAD study, reference [13,14].

*** Intensity ratio (%) = (Intensity with SPE/ Intensity without SPE) x 100, where intensities are in positive mode.

### Effect of SPE conditions on detection pattern

The effect of SPE conditions on the detection pattern of 27 detected substances was investigated by comparison with the results without SPE in the positive control (Table 5). The acidic SPE condition showed the highest retention among the three conditions with two undetectable substances (AM-6 and IM-10), followed by basic SPE with five undetectable substances (AM-8, CPM-3, CTM-3, CTM-5 and CTM-7), and neutral SPE with six undetectable substances (AM-8, IM-10, CPM-3, CTM-3, CTM-5 and CTM-7). 

Major unique metabolites, e.g. AM-2, IM-1 and CM-1, were retained well by three SPE conditions used. Almost all of the common acidic metabolites, e.g. CPM-3 and CTM-3, were not retained well by basic and neutral SPE conditions. Basic metabolites, e.g. AM-6 and IM-10, were not retained well by acidic SPE condition, whereas some metabolites, e.g. AM-8, CM-2 and CM-7, were not retained well by basic SPE condition.

### Qualitative profiling of neonicotinoid metabolites in the human urine

Eight out of 27 metabolites were detected in the human urine from the three patients (#1, #2, and #3) (Table 6, Figure 1). Acetamiprid was found in Case #2, *N*-desmethyl-acetamiprid (AM-2) in Cases 1 and 3, and *N*-(6-chloronicotinoyl)-glycine (CPM-8) in the all three cases. 5-Hydroxy-imidacloprid (IM-1) was found in Cases #2 and #3, 4,5-dihydroxy-imidacloprid (IM-3) in the all three cases, and 4,5-dehydro-imidacloprid (IM-7) in Case #1. *N*-desmethyl-clothianidin (CM-1) was found in Case #2 and *N*-(2-(methylsulfanyl)thiazole-5-carboxyl)-glycine (CTM-8) was found in the all three cases. Interestingly, the precursors of CPM-8 and CTM-8, i.e. 6-chloronicotinic acid (CPM-3) and 2-chlorothiazole-5-carboxilic acid (CTM-3), were not detected in the three human urine samples. 

**Table 6:** Mean intensities of neonicotinoid pesticide metabolites detected by LC/TOFMS and concentrations determined by LC/MS/MS in the urine of three cases.

**Case**	**Name**	**SPE condition/Ionization mode**						**LC/TOFMS**	**Conc.**
		**Acid/+**	**Acid/-**	**Neutral/+**	**Neutral/-**	**Base/+**	**Base/-**	**Decision**	**(ng/mL)**
#1	Acetamiprid	23	39	0	0	0	0	<LOQ	0.058 *
	AM-2	807	246	1028	700	1191	840	Detected	3.2
	CPM-8	0	30956	0	1027	0	0	Detected	N.D. **
	IM-1	73	11	32	31	24	49	<LOQ	N.D.
	IM-3	0	401	0	0	0	0	Detected	N.D.
	IM-7	11	649	0	596	62	606	Detected	N.D.
	CM-1	0	19	0	5	0	17	<LOQ	N.D.
	CTM-8	0	4444	0	13	0	6	Detected	N.D.
#2	Acetamiprid	303	0	193	0	217	0	Detected	< LOD
	AM-2	7	0	0	0	0	0	<LOQ	< LOD
	CPM-8	0	5128	0	3111	0	0	Detected	N.D.
	IM-1	436	0	410	0	345	6	Detected	N.D.
	IM-3	0	658	0	0	0	0	Detected	N.D.
	IM-7	0	29	5	60	0	0	<LOQ	N.D.
	CM-1	0	195	0	100	0	95	Detected	N.D.
	CTM-8	0	2449	12	0	0	0	Detected	N.D.
#3	Acetamiprid	0	5	0	0	0	0	<LOQ	< LOD
	AM-2	252	8	148	46	181	70	Detected	0.48 *
	CPM-8	0	8529	0	1102	0	0	Detected	N.D.
	IM-1	458	0	220	0	133	0	Detected	N.D.
	IM-3	0	914	0	0	0	6	Detected	N.D.
	IM-7	16	34	0	30	0	0	<LOQ	N.D.
	CM-1	0	0	0	0	0	0	<LOQ	N.D.
	CTM-8	0	167	0	0	0	0	Detected	N.D.

LOD and LOQ stand for the lowest level of detection and the lowest level of quantification, respectively.

* Less than LOQ, ** N.D. stands for not determined.

### Quantification of acetamiprid and *N-*desmethyl-acetamiprid (AM-2) in the human urine

The standard curves for acetamiprid and AM-2 are linear between the highest and the lowest concentrations. The linear regression equation for acetamiprid is y = 1.8636x + 0.1263 (R^2^ = 0.9995), and y = 0.4887x + 0.1996 (R^2^ = 0.9946) for AM-2. The lowest level of quantification (LOQ) for acetamiprid and AM-2 are 0.068 and 0.55 ng/mL, respectively. The recovery efficiencies for acetamiprid and AM-2 are 100.3 ± 7.7 % and 97.7 ± 5.2 %, respectively. 

**Figure 3:**
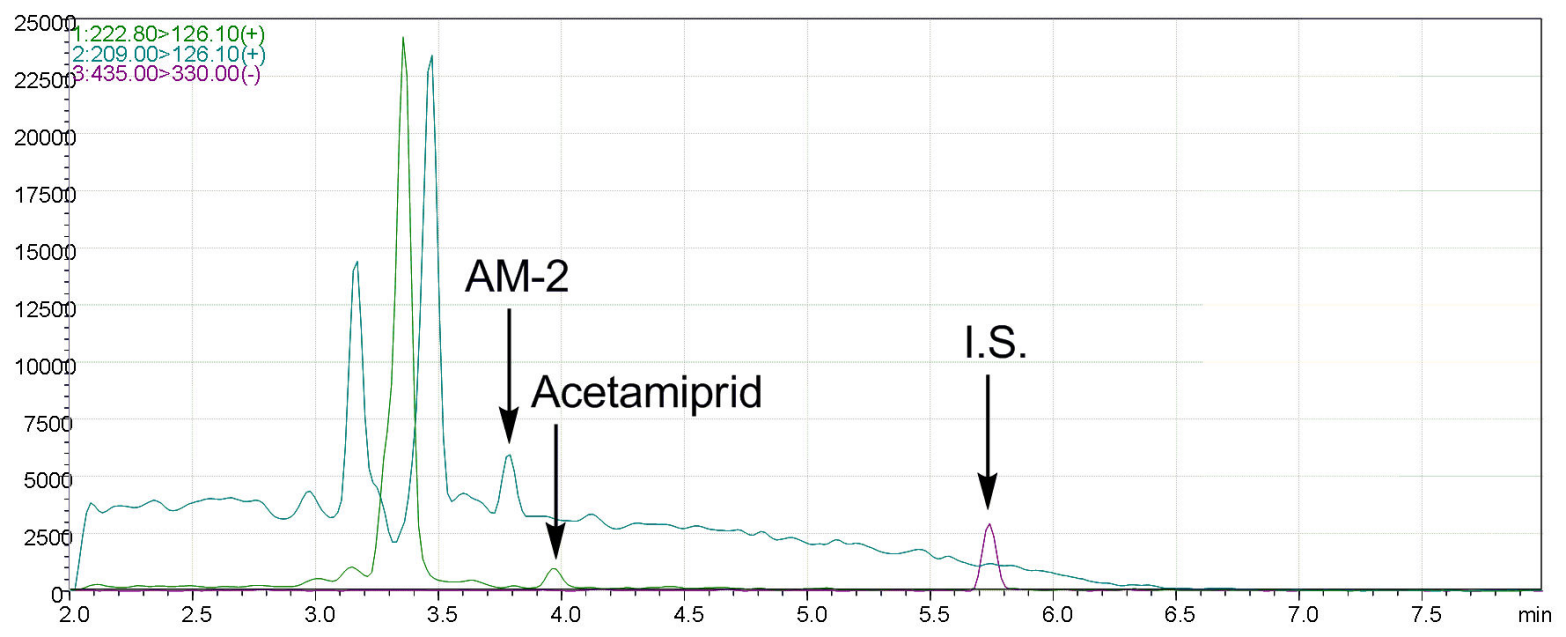
LC/MS/MS of the extract of standard urine solution containing acetamiprid (green), *N*-desmethyl-acetamiprid (AM-2) (blue) and internal standard (I.S.) (red).

**Figure 4:**
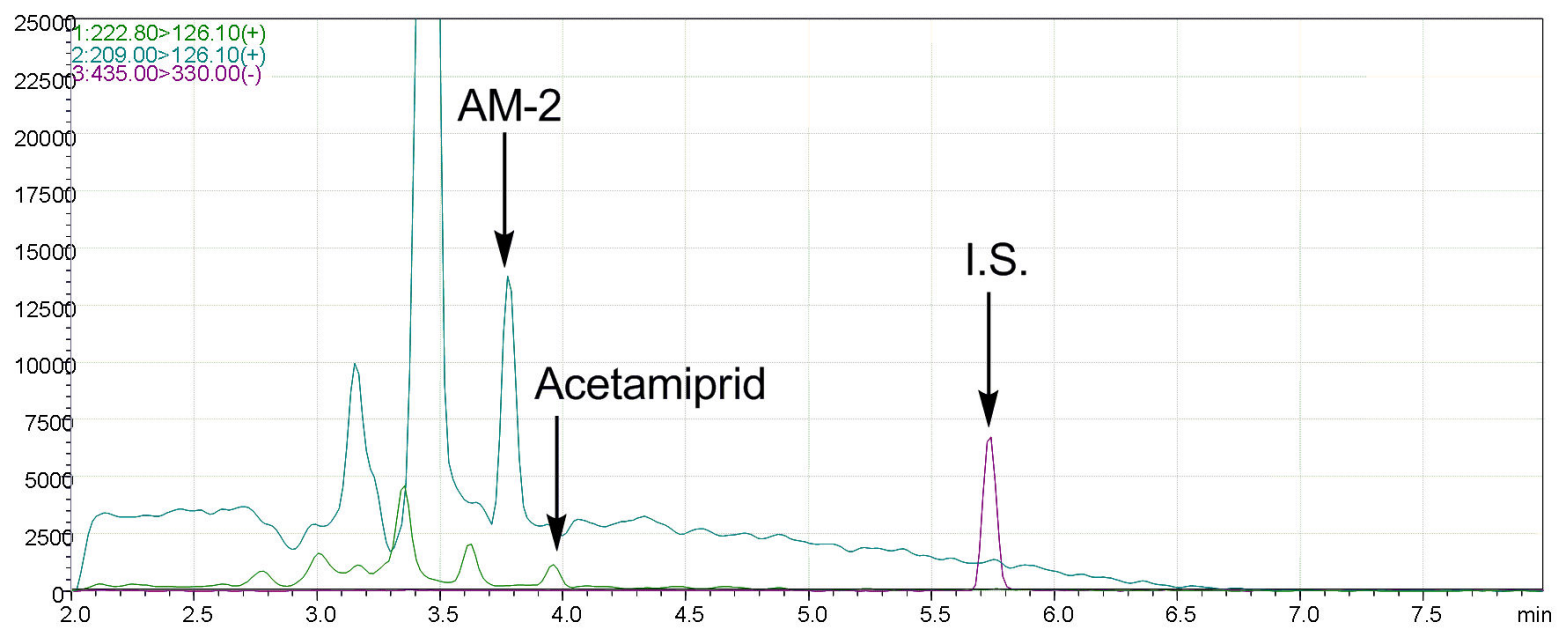
LC/MS/MS of the extract of urine of one case (#1) containing acetamiprid (green), *N*-desmethyl-acetamiprid (AM-2) (blue) and internal standard (I.S.) (red).

AM-2 was detected in the urine of Case #1 at a concentration of 3.2 ng/mL. Acetamiprid was not detected in Case #1 urine at more than LOQ. Representative LC/MS/MS of extracts from a standard solution and a human urine sample are shown in Figures 3 and 4, respectively. AM-2 or acetamiprid was not detected from the urine of Case #2 who recovered more rapidly than Case #1. AM-2 or acetamiprid was also not detected in the urine of Case #3 at more than LOQ who had already recovered after stopping the high dose intake of tea or fruits (Table 6). From the seven urine samples of the negative control group (#4-10), AM-2 or acetamiprid was not detected at more than LOQ (Table 2). 

## Discussion

This research showed, for the first time, *N*-desmethyl-acetamiprid (AM-2) was qualitatively detected and quantified in the human urine. The concentration of urinary AM-2 in Case #1, 3.2 ng/mL, is equivalent to 0.015 μM. In literature, the blood concentration of acetamiprid in 3 cases of acute severe poisoning was 2.39–59.83 μg/mL (10.7–269 μM), whereas that of imidacloprid in 7 cases of acute poisoning was 2.05–12.5 μg/mL (9.21–56.1 μM) in lethal cases, and 3.0–84.9 ng/mL (0.013–0.381 μM) in severe cases [1,9,10,12]. The urinary AM-2 level of Case #1 is within the range of plasma imidacloprid levels in severe cases. Although the relationship between urinary AM-2 levels and plasma acetamiprid levels is unknown, it can be concluded that Case #1 had an exposure to acetamiprid at a toxic level. The high-level excretion of AM-2 in Case #1 may be caused by an exceptional high-level contamination of acetamiprid in food and/or tea beverages that she had continuously consumed or by her specific metabolic condition. Furthermore, Case #1 may be exposed to other neonicotinoid pesticides, which were not scrutinized in this study. The quantitative analysis of other neonicotinoid metabolites in human urine by LC/MS/MS needs to be investigated.

It is still unknown whether the low-level oral exposure to neonicotinoid would cause nicotinic symptoms or not. The accumulation of acetamiprid in mammals has never been reported, whereas the half-life of acetamiprid is relatively longer than other neonicotinoids [14]. 

Case #1 had been healthy two months prior to the onset, and began consuming 1000–2000 mL of Oolong tea daily. She drank grape juice during the last 10 days. She ate a domestic Asian pear at 7 am, and felt a sense of discomfort 30 minutes later, followed by nasal obstruction, throat constriction, generalized muscle pain, and stomachache one hour later. When she visited a-clinic at 9 am, she was drowsy (JCS1), showed high fever (39.8 °C), cervical lymph node swelling, SpO_2_ 98 %, short-term memory disturbance, muscle spasm and marked finger tremor. Elevation in CRP (1.12 mg/dL), neutrophilia (9590/μL), lymphocytopenia (1190/μL) and diminution in basophil and eosinophil (12/μL each) was observed by a blood-examination finding. Intermittent WPW syndrome at a rate of 1/1 (110 bpm) was observed by electrocardiography. By the next day, her stomachache, headache, muscle pain, and arrhythmia had improved. We detected urinary 6-chloronicotinic acid (CPM-3) from Case #1 on the second day after the first visit at a concentration of 59.1 ng/mL by LC/MS [11]. 

If the AM-2 level of the spot urine of Case #1 is at a steady state, the daily excretion of AM-2 is calculated to be 3.2–4.8 μg. And if urinary AM-2 consists of 10 % of exposed dose of acetamiprid, the daily intake of acetamiprid is estimated to be 34–51 μg, or 0.68–1.02 μg/kg. The estimated daily intake is 69-fold to 104-fold less than the Acceptable Daily Intake (ADI) for acetamiprid (0.071 mg/kg) [28]. The low estimated intake may be due to over-estimation of urinary AM-2 to the amount of intake, or the lower AM-2 level in spot urine than that in plasma. 

The results of this study suggest that the universal use of neonicotinoids would cause the unintended exposure to neonicotinoids in children who are more sensitive to neurotoxicants because of their neural development [29]. We reported a case of an 8-year-old girl who exhibited nicotinic symptoms with an electrocardiographic change and a detection of CPM-3 in her urine [11]. There are some reports that indicate the significant association between the conventional fruits/vegetable intake and the urinary pesticide metabolites, for example, tomato consumption and urinary concentrations of a metabolite of pyrethroid pesticides, 3-phenoxybonzoic acid, in the general population in Japan [30]. Another report showed that the median urinary concentrations of metabolites of organophosphorus pesticides, i.e. malathion dicarboxylic acid for malathion and 3,5,6-trichloro-2-pyridinol for chlorpyrifos, are significantly correlated with the median consumption of fresh product (fruits, juices, and vegetables) in children [31]. Children with conventional diets reportedly had significantly higher levels of urinary organophosphorus metabolites, dimethyl alkylphosphates, than did children with organic diets [32]. Epidemiological studies on urinary neonicotinoid metabolites are needed to establish their reference levels in combination with food consumption in the general population.

In this study, 27 substances were detected in the positive controls by LC/TOFMS. Other authors have reported 43 substances in the urine of the male albino Swiss-Webster mice by LC/MS and HPLC/DAD methods after acute high dose exposure [13,14]. Although the number of substances detected in our study was smaller than those studies, two substances, i.e. AM-8 (N-[(6-chloropyridin-3-yl)methyl]formamide) and IM-2 (1-(6-chloro-3-pyridylmethyl)-2-nitroguanidine), were detected and reported for the first time. The difference in the profile of detected metabolites may be attributed to the difference in analytical methodologies, as well as in mouse strains. The top three metabolites in the urine from each neonicotinoid-dosed mouse were as follows: AM-2, CPM-5 and CPM-8 for acetamiprid; IM-1, IM-3 and IM-7 for imidacloprid; and CM-1, CM-2 and CM-4 for clothianidin. The results are in accord with the previously reported urinary metabolite profile in experimental animals [13,14], and the human urinary metabolite detected in this study. Our results indicate that major neonicotinoid metabolites in the urine of experimental animals, such as AM-2, IM-1 and CM-1, can be used as urinary biomarkers for humans. 

The limitation of this study is that we chose the urine of mice that were administered neonicotinoids intra-peritoneally as the positive control, because recent papers had showed it developed major metabolites [13,14]. It is known that there is a significant diversity of neonicotinoid metabolism in species, as well as the activity of enzymes, e.g. cytochrome P450 and aldehyde oxidase [33]. Other neonicotinoid metabolites may be detectable in the urine of other species, such as rabbits, after oral administration of neonicotinoid. 

The limitation of the LC/MS/MS method of AM-2 is high LOQ, which is 8-fold higher than that of acetamiprid, because of its high instrumental detection limit. Although there is a possibility of false detection of AM-2, it appears to be low because the retention time was identical with the standard compound by LC/MS/MS with two different stationary phases. Nevertheless the finding of this study is important for the future study, because AM-2 seems to be a good and specific biomarker for acetamiprid exposure in humans. The maximum AM-2 level was at least 55-fold higher than that of acetamiprid. Therefore, even though the sensitivity is 8-fold less than acetamiprid, the chance to detect urinary AM-2 appears to be higher. 

## Conclusions

A qualitative profiling by LC/TOFMS led to the qualitative detection of seven metabolites, including *N*-desmethyl-acetamiprid (AM-2), 5-hydroxy-imidacloprid (IM-1) and *N*-desmethyl-clothianidin (CM-1), and acetamiprid in some human urine samples. A subsequent LC/MS/MS analysis confirmed that AM-2 was quantified in the urine of one case at a level of 3.2 ppb, suggesting that the person consumed foods or beverage contaminated with high level of acetamiprid. This is the first report of quantitative detection of AM-2 in the urine of a patient, who is suspected of subacute neonicotinoid intoxication through consumption of contaminated food. AM-2 appears to be a useful urinary biomarker for acetamiprid exposure in humans. This study is the first step toward the development of urinary biomarkers for neonicotinoid pesticides and warrants further investigations of neonicotinoid exposure in the general population. 

## Supporting Information

Table S1 **The names, common names, alternative abbreviations in literature and oral toxicity of seven neonicotinoid pesticides and metabolites.**
(DOCX)
