# Correction: Qualitative Profiling and Quantification of Neonicotinoid Metabolites in Human Urine by Liquid Chromatography Coupled with Mass Spectrometry

**DOI:** 10.1371/annotation/af11d412-0748-4ecd-98de-0d75e4ce0eaf

**Published:** 2014-01-23

**Authors:** Kumiko Taira, Kazutoshi Fujioka, Yoshiko Aoyama

The chemical structure of clothianidin and N-desmethyl-clothianidin (CM-1) in Figure 1 is incorrect. The correct version of Figure 1 can be viewed here: http://plosone.org/corrections/pone.0080332.g001.cn.tif

