# Supplementary material for: Qualitative Profiling and Quantification of Neonicotinoid Metabolites in Human Urine by Liquid Chromatography Coupled with Mass Spectrometry
Source: PLoS One. 2013 Nov 12;8(11):e80332. doi: 10.1371/journal.pone.0080332 (PMC3827204; doi:10.1371/journal.pone.0080332)
Supplement: Table S1 — The names, common names, alternative abbreviations in literature and oral toxicity of seven neonicotinoid pesticides and metabolites. (DOCX) [file pone.0080332.s001.docx]

Table S1. The names, common names, alternative abbreviations in literature and oral toxicity of seven neonicotinoid pesticides and metabolites

| **Name** | **Common name** | **Alternative** | **Oral LD50 (mg/kg BW)** | |
| --- | --- | --- | --- | --- |
| **used in this study** |  | **abbreviation**** | **in rodents***** | |
|  |  |  | **male** | **female** |
| Acetamiprid | Acetamiprid | ACE | 198-217 | 146-184 |
| AM-1 | *N*-((6-Chloropyridin-3-yl)-methyl)-*N*-methyl-acetamide | ACE-acet | 1142 | 900-1000 |
| AM-2 | *N*-Desmethyl-acetamiprid | ACE-dm | 2543 | 1276 |
| AM-3 | *N*-((6-Chloropyridin-3-yl)-methyl)- acetamide | ACE-dm-acet | 1378 | 900-1000 |
| AM-4 |  | ACE-dm-NCONH2 |  |  |
| AM-5 |  | ACE-U |  |  |
| AM-6 | *N*-((6-Chloropyridin-3-yl)-methyl)-methylamine | CNCP-r | 1224-1259 | 963-1176 |
| AM-7 | *N*-(6-Chloropyridin-3-yl)-methylamine | CNCP-s | 1592 | 1381 |
| AM-8 | *N*-(6-Chloropyridin-3-yl)-formamide | CNCP-t |  |  |
| AM-9 | *N*-Cyano-*N*’-methyl-acetamidine | CNCP-u | 2662 | 2420 |
| AM-10 |  | CNCP-v |  |  |
| AM-11 | *N*-Cyano-acetamidine | CNCP-w | >5000 | >5000 |
| AM-12 | *N*-Decyano-acetamiprid | ACE-NH | 141-195 | 132-200 |
| AM-13 |  | ACE-NCONH_2_ |  |  |
| Imidacloprid | Imidacloprid | IMI | 100-440 | 98-475 |
| IM-1 | 5-Hydroxy-imidacloprid | IMI-5-OH |  |  |
| IM-2 |  | IMI-de |  |  |
| IM-3 | 4,5-Dihydroxy-imidacloprid | IMI-diol |  |  |
| IM-4 | *N*-Denitro-imidacloprid | IMI-NH | 300 | 280 |
| IM-5 |  | IMI-NNH2 |  |  |
| IM-6 | *N*-((6-Chloropyridin-3-yl)-methyl)-*N*’-nitroso-imidazolidin-2-ylideneamine | IMI-NNO | 200-1980 | 200-3560 |
| IM-7 | 4,5-Dehydro-imidacloprid | IMI-ole | 3500 | 1100 |
| IM-8 |  | IMI-tri |  |  |
| IM-9 | *N*-((6-Chloropyridin-3-yl)-methyl)-imidazolidinone | IMI-urea | 4080 | 1820 |
| IM-10 |  | CNCP-l |  |  |
| IM-11 |  | CNCP-m |  |  |
| IM-12 |  | IMI-5-OH-gluc |  |  |
| IM-13 |  | IMI-urea-gluc |  |  |
| IM-14 |  | IMI-urea-gent |  |  |
| Clothianidin | Clothianidin | CLO | 389->5000 | 465->5000 |
| CM-1 | *N*-Desmethyl-clothianidin | CLO-dm |  | 1480 |
| CM-2 | *N*-Desmethyl-*N‘*-denitro-clothianidin | CLO-dm-NH |  |  |
| CM-3 |  | CLO-dm-NNH2 |  |  |
| CM-4 |  | CLO-dm-NNO |  |  |
| CM-5 |  | CLO-dm-tri |  |  |
| CM-6 |  | CLO-dm-urea |  |  |
| CM-7 | *N*-Denitro-clothianidin | CLO-NH |  |  |
| CM-8 |  | CLO-NNH2 |  |  |
| CM-9 |  | CLO-NNO |  |  |
| CM-10 |  | CLO-tri |  |  |
| CM-11 | *N*-(2-Chlorothiazole-5-methyl)-*N*’-methyl-urea | CLO-urea | 1420 | 1280 |
| CM-12 | *N*-methyl-*N*’-nitroguanidine | NG-E |  |  |
| CM-13 | *N*-methyl-guanidine | NG-F | 550 | 446 |
| CPM-1 | 6-Chloronicotinic aldehyde | CNCP-a |  |  |
| CPM-2 | 6-Chloropyridine-3-methanol | CNCP-b (CPOL) | 1842, 3800 | 1483, 3700 |
| CPM-3 | 6-Chloronicotinic acid | CNCP-c (CPCA) | >5000 | >5000 |
| CPM-4 |  | CNCP-d |  |  |
| CPM-5 | 2-Methylsulfanylpyrydin-5-yl-carboxilic acid | CNCP-e |  |  |
| CPM-6 |  | CNCP-f |  |  |
| CPM-7 | 2-Hydroxypyridin-5-yl-carboxylic acid | CNCP-g (6-OH-PCA) |  |  |
| CPM-8 | *N*-(6-Chloronicotinoyl)-glycine | CNCP-h |  |  |
| CPM-9 |  | CNCP-i |  |  |
| CPM-10 |  | CNCP-j |  |  |
| CPM-11 | Methyl 6-chloronicotinate | CPCA-Me |  |  |
| CPM-12 | 2-Mercaptopyrydin-5-yl-carboxilic acid | 6-HS-PCA |  |  |
| CPM-13 | 2-Pyridone | pyridone |  |  |
| CPM-14 | *N*-(6-Chloronicotinoyl)-alanine | CPCA-Ala |  |  |
| CPM-15 | *N*-(6-Chloronicotinoyl)-aspartic acid | CPCA-ASP |  |  |
| CPM-16 | *N*-(6-Chloronicotinoyl)-cysteine | CPCA-Cys |  |  |
| CPM-17 | *N*-(6-Chloronicotinoyl)-leucine | CPCA-Leu |  |  |
| CPM-18 | *N*-(6-Chloronicotinoyl)-phenylalanine | CPCA-Phe |  |  |
| CPM-19 | *N*-(6-Chloronicotinoyl)-proline | CPCA-Pro |  |  |
| CPM-20 | *N*-(6-Chloronicotinoyl)-threonine | CPCA-Thr |  |  |
| CPM-21 | *N*-(6-Chloronicotinoyl)-valine | CPCA-Val |  |  |
| CPM-22 | *N*-(2-Hydroxypyridin-5-yl-carboxyl)-aspartic acid | 6-OH-PCA-Asp |  |  |
| ICM-1 | Nitroguanidine | CNCP-k (NG-G) * | 3120-10200 | 3120-10200 |
| CTM-1 | 2-Chlorothiazole-5-carboxaldehyde | CTM-a |  |  |
| CTM-2 | 2-Chlorothiazole-5-methanol | CTM-b |  |  |
| CTM-3 | 2-Chlorothiazole-5-carboxilic acid | CTM-c (CTCA) |  |  |
| CTM-4 |  | CTM-d |  |  |
| CTM-5 |  | CTM-e |  |  |
| CTM-6 | *N*-2-Methylsulfanylthiazole-5-carboxylic acid | CTM-f |  |  |
| CTM-7 | *N*-(2-Chlorothiazole-5-carboxyl)-glycine | CTM-g |  |  |
| CTM-8 | *N*-(2-(Methylsulfanyl)thiazole-5-carboxyl)-glycine | CTM-h |  |  |
| CTM-9 | 2-Chlorothiazole-5-methylamine | CTM-i |  |  |
| CTM-10 | *N*-Acetyl-2-chlorothiazole-5-methylamine | CTM-j |  |  |
| Thiacloprid | Thiacloprid | THI | 127-836 | 147-444 |
|  | *N*-Decyano-thiacloprid | THI-NH |  | 1.1-28 |
|  | 4,5-Dehydro-thiacloprid | THI-ole-NH |  |  |
|  | 4-Hydroxy- thiacloprid | THI-4-OH |  |  |
|  |  | THI-NCONH_2_ |  |  |
|  |  | THI-4-OH-NCONH2 |  |  |
|  |  | THI-SO |  |  |
|  |  | THI-SO_3_H-NCONH_2_ |  |  |
|  |  | THI-SOMe |  |  |
| Nitenpyram | Nitenpyram | NIT | 867-1680 | 1281-1575 |
|  | *N*-Desmethyl-nitenpyram | NIT-dm |  |  |
|  |  | NIT-dm-COOH |  |  |
|  |  | NIT-CN |  |  |
|  |  | NIT-dm-de |  |  |
|  |  | NIT-desCN |  |  |
| Thiamethoxam | Thiamethoxam | TMX | 783-1563 | 964-1563 |
|  |  | TMX-NNO |  |  |
|  |  | TMX-NNH_2_ |  |  |
|  | *N*-Denitro-thiamethoxam | TMH-NH |  |  |
|  |  | TMX-urea |  |  |
|  |  | NG-A |  |  |
|  |  | NG-B |  |  |
|  | *N*-Desmethyl-thiamethoxam | TMX-dm |  |  |
|  |  | TMX-dm-NNO |  |  |
|  |  | TMX-dm-NNH_2_ |  |  |
|  |  | TMX-dm-tri |  |  |
|  | *N*-Desmethyl-*N‘*-denitro-thiamethoxam | TMX-dm-NH |  |  |
|  |  | TMX-dm-urea |  |  |
|  |  | NG-C |  |  |
|  |  | NG-D |  |  |
| Dinotefuran | Dinotefuran | DIN | 2450-2804 | 2000-2275 |
|  |  | DIN-NNO |  |  |
|  |  | DIN-NNH_2_ |  |  |
|  |  | DIN-tri |  |  |
|  | *N*-Denitro-dinotefuran | DIN-NH |  |  |
|  | *N*-(3-Furayl)-*N‘*-methylurea | DIN-urea |  |  |
|  | *N*-Desmethyl-dinotefuran | DIN-dm |  |  |
|  |  | DIN-dm-NNO |  |  |
|  |  | DIN-dm-NNH_2_ |  |  |
|  |  | DIN-dm-tri |  |  |
|  | *N*-Desmethyl-*N‘*-denitro-dinotefuran | DIN-dm-NH |  |  |
|  | 2-Hydroxy-dinotefuran | DIN-2-OH |  |  |
|  |  | DIN-a |  |  |
|  |  | DIN-b |  |  |
|  |  | DIN-c |  |  |
|  |  | DIN-d |  |  |
|  |  | DIN-e |  |  |
|  |  | DIN-f |  |  |
|  |  | DIN-4-OH |  |  |
|  |  | DIN-5-OH |  |  |
|  |  | DIN-g |  |  |
|  |  | DIN-h |  |  |
|  |  | DIN-i |  |  |
|  |  | DIN-j |  |  |
|  |  | DIN-k |  |  |
|  | 3-Furfural | DIN-l |  |  |
|  | 3-Furfuryl alcohol | DIN-m (THFOL) |  |  |
|  | Tetrahydrofuran-3-carboxylic acid | DIN-n (THFCA) |  |  |
|  | *N*-(Tetrahydrofuran-3-carboxyl)-glycine | DIN-o |  |  |
|  | 4-Hydroxy-tetrahydrofuran-3-carboxylic acid | DIN-p |  |  |
|  | *N*-(4-Hydroxy-tetrahydrofuran-3-carboxyl)-glycine | DIN-q |  |  |
|  | Tetrahydrofuran-3-yl-methylamine | DIN-r (THFMA) |  |  |
|  | *N*-(Acetyl-tetrahydrofuran-3-yl-methylamine | DIN-s |  |  |

*: 2-Nitroguanidine (CNCP-k) and 1-nitroguanidine (NG-G) are tautomeric forms of nitroguanidine and freely convert to each other in solutions. **: Reference [13-15]. ***: Reference [16-21]
